# Development of a fluorescence and quencher-based FRET assay for detection of endogenous peptide:*N*-glycanase/NGLY1 activity

**DOI:** 10.1016/j.jbc.2024.107121

**Published:** 2024-02-28

**Authors:** Hiroto Hirayama, Yuriko Tachida, Reiko Fujinawa, Yuka Matsuda, Takefumi Murase, Yuji Nishiuchi, Tadashi Suzuki

**Affiliations:** 1 Glycometabolic Biochemistry Laboratory, RIKEN Cluster for Pioneering Research (CPR), Riken, Wako Saitama, Japan; 2 Takeda-CiRA Joint Program (T-CiRA), Fujisawa, Kanagawa, Japan; 3 Glytech. Inc., Shimogyo-ku, Kyoto, Japan

**Keywords:** peptide:N-glycanase, endo-β-N-acetylglucosaminidase, FRET, enzyme assay, aging

## Abstract

Cytosolic peptide:*N*-glycanase (PNGase/NGLY1 in mammals) catalyzes deglycosylation of *N*-glycans on glycoproteins. A genetic disorder caused by mutations in the NGLY1 gene leads to NGLY1 deficiency with symptoms including motor deficits and neurological problems. Effective therapies have not been established, though, a recent study used the administration of an adeno-associated viral vector expressing human NGLY1 to dramatically rescue motor functions in young *Ngly1*^*−/−*^ rats. Thus, early therapeutic intervention may improve symptoms arising from central nervous system dysfunction, and assay methods for measuring NGLY1 activity in biological samples are critical for early diagnostics. In this study, we established an assay system for plate-based detection of endogenous NGLY1 activity using a FRET-based probe. Using this method, we revealed significant changes in NGLY1 activity in rat brains during aging. This novel assay offers reliable disease diagnostics and provides valuable insights into the regulation of PNGase/NGLY1 activity in diverse organisms under different stress conditions.

Cytosolic peptide:*N*-glycanase (PNGase/NGLY1 in mammals) is a widely conserved eukaryotic amidase (EC 3.5.1.52) that catalyzes the de-*N*-glycosylation of glycoproteins and the conversion of Asn to Asp residues within an *N*-glycosylated Asn-Xaa-Ser/Thr consensus sequence (where Xaa is any amino acid except proline) (1). The cytosolic PNGase-dependent deglycosylation of misfolded glycoproteins can facilitate their proteasomal degradation by removing bulky *N*-glycans that obstruct the accessibility of substrates to proteasome active sites (2). More recently, a unique aspect of the glycosylated-Asn-to-Asp editing activity of NGLY1 was reported (3, 4), in which the editing of a transcription factor, nuclear factor erythroid2-like 1 also known as nuclear factor erythroid2-related factor 1 in mammals and SKN-1A in *Caenorhabditis elegans*, plays a critical role. Editing is required for activation of this transcription factor (3, 4, 5, 6), thus, demonstrating the multifunctionality of this protein. The *N*-glycans released by NGLY1 in the cytosol undergo additional processing by cytosolic glycosidases, including endo-β-*N*-acetylglucosaminidase (ENGase) and α-mannosidase (Man2C1) (7, 8). Cytosolic ENGase also functions as a deglycosylating enzyme that targets *N*-glycans on misfolded glycoproteins and converts them into single *N*-GlcNAc residues (9, 10, 11, 12).

An autosomal-recessive disorder linked to NGLY1, designated as NGLY1 deficiency or congenital disorder of deglycosylation (NGLY-CDDG) [OMIM: 615273], was reported in 2012 (13). Since then, over 100 patients have been reported worldwide in Europe, America, Australia, India, China, and Japan (14, 15). Disease symptoms are broad and include global developmental delay and/or intellectual disability, abnormal electroencephalogram, seizure, movement disorder, hypo- or alacrima, and liver dysfunction (13, 15, 16, 17, 18, 19, 20, 21). Although the detailed molecular mechanisms of disease pathophysiology remain unclear, it is known that NGLY1 is essential for cytosolic misfolded protein deglycosylation, activation of a transcription factor, nuclear factor erythroid2-like 1 (3, 4, 5, 6, 22), homeostasis of mitochondria (23), regulation of mitophagy (24), innate immunity related to the cyclic GMP-AMP synthase (cGAS)–stimulator of interferon genes (STING) pathway (24), AMP signaling pathway (25), BMP signaling (26, 27), and neuron development (28, 29). Unfortunately, effective therapeutics have yet to be established, although enzyme replacement or gene therapy in the central nervous system (CNS) should be promising for disease treatment, as reported for other lysosomal-storage diseases. This presumption is partially supported by recent reports that *Ngly1*^*−/−*^ rats exhibit various defects in motor functions and that intracerebroventricular administration of an adeno-associated virus 9 vector carrying human *NGLY1* into young *Ngly1*^*−/−*^ rats alleviates various motor dysfunctions (30, 31, 32). These observations indicate that early therapeutic intervention should improve various symptoms arising from CNS dysfunction in this disease. Therefore, methods are urgently needed for the early diagnosis of NGLY1 deficiency by measuring endogenous NGLY1 activity in specimens from disease candidates.

Many biochemical methods for measuring PNGase/NGLY1 activity have been reported, and two primary methods are used. In the first assay, fetuin-derived ^14^C-labeled glycopeptides are used as substrates, and enzyme activity is measured via autoradiography after separation by paper chromatography or paper electrophoresis (33, 34, 35). However, this method is unsuitable for a normal laboratory because of challenges in the radiolabeled substrate preparation and tight regulations regarding the use of radioisotopes. A different assay uses *S*-alkylated RNaseB as a substrate in a reaction with an enzyme source, and the substrate and product are then separated by SDS-PAGE and detected by immunoblot or Coomassie brilliant blue staining (36). However, this assay is incompatible with measurements in crude enzyme samples because substrates are degraded by contaminating endogenous peptidases. Therefore, it is imperative to establish a simple and sensitive method for the measurement of endogenous NGLY1 activity in various enzyme sources.

In our previous study, we established an assay system using 5-carboxyfluorescein-labeled glycosylated hepta-cyclopeptide (5FAM-GCP), which is highly resistant to endogenous peptidases (37, 38). This method allows us to measure endogenous NGLY1 activity in a wide variety of samples (*e.g.*, cell lines, tissues, peripheral blood mononuclear cells (PBMC), and patients' cells) without any proteolytic degradation of the substrate and the product. However, this assay uses HPLC for product separation and detection, which is often not available for standard clinical laboratories. It is, therefore, crucial to develop a straightforward and highly sensitive method for enzyme assay, such as MANT-M3GN2-DNP (MM3D), a fluorescence and quencher-based FRET probe designed for detecting ENGase activity (39). In this study, we demonstrate that a novel FRET-based GCP probe (fGCP), which is composed of a glycan modified with fluorophore-labeled bisected-GlcNAc (AMCA-GlcNAc) and a cyclopeptide modified with a Dabcyl quencher, can detect endogenous NGLY1 activity in various enzyme sources via fluorescence on multiarray plates. Using this method, we observed significant changes in the levels of Ngly1 activity in aging rat brains. Our novel assay method would not only offer a reliable diagnostic tool but also provide valuable insights into the regulation of PNGase/NGLY1 activity in diverse organisms under different stress conditions.

## Results

### Optimization of the glycan structure on GCP for the construction of a novel FRET-based NGLY1 probe

**Figure 1:**
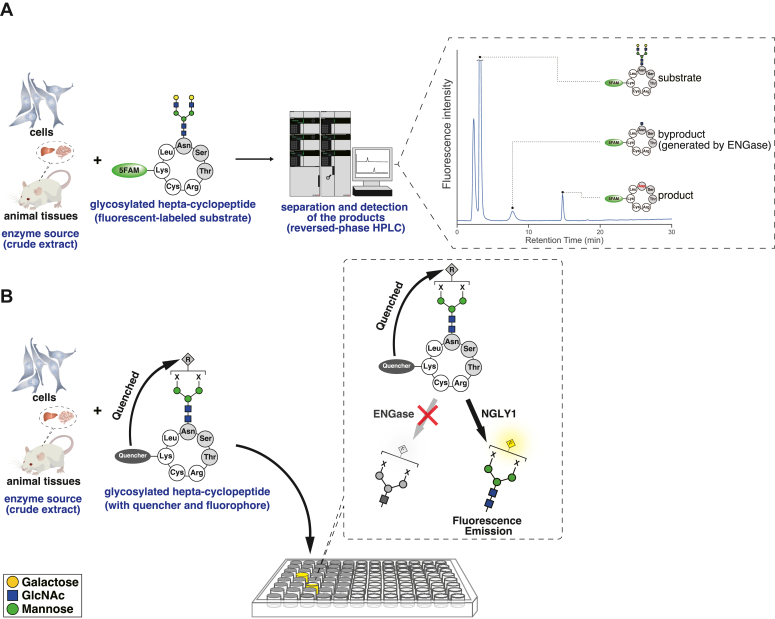
**Strategy for the detection of NGLY1 activity using a FRET-based probe.***A*, the measurement of NGLY1 (PNGase) activity using 5FAM-GCP and HPLC. The HPLC chart represents separation pattern of the substrate (5FAM-GCP), byproduct (5FAM-GCP with mono-GlcNAc), and product (PNGase-deglycosylated 5FAM-GCP) in reversed-phase HPLC. *B*, a FRET-based probe for the detection of NGLY1 activity. R in a *gray diamond* and F in a *yellow diamond* represent extinction and emission of fluorescence, respectively. X at the nonreducing end of *N*-glycans represents glycan structures that are substrates for NGLY1 and resistant to ENGase. 5FAM-GCP, 5-carboxyfluorescein-labeled GCP; GCP, glycosylated hepta-cyclopeptide.

In our previous study, we demonstrated that an assay using glycosylated hepta-cyclopeptide (GCP) as a substrate enabled us to measure endogenous NGLY1 activity in crude extracts prepared from various cells and tissues, including rodent tissues, cell culture lines, iPS cells, and PBMCs, without any substrate degradation by endogenous proteases/peptidases (Fig. 1*A*) (37, 38). This substrate, however, tends to be processed by the cytosolic ENGase and is converted to *N*-GlcNAc GCP as a byproduct (Fig. 1*A*). This issue must be resolved to evolve GCP into a FRET-based GCP probe in which the cyclopeptide moiety is modified with fluorophore-labeled glycan and quencher. To overcome this problem, we sought to identify glycan structures that are sensitive to NGLY1 activity but highly resistant to ENGase activity (Fig. 1*B*). Specifically, the substrate specificity of purified recombinant human ENGase was examined using several structures of pyridylamino-labeled (PA-labeled) glycans as substrates. As shown in Table 1, human ENGase prefers high-mannose-type glycans (Man6 and Man9 forms) over complex-type glycans (agalacto-biantennary glycan; A2 form). Additionally, this enzyme exhibits no detectable activity toward tri/tetra-branched glycans (A3 and A4 forms), a core fucosylated glycan (FA2 form), or an A2 glycan modified with bisected-GlcNAc (A2B form). Some properties of human ENGase are consistent with previous reports that several cytosolic ENGases categorized in the GH85 family also exhibit no activity against tri- and tetra-branched complex glycans (40, 41, 42).

**Table 1:** Relative activity of human ENGase for various structures of *N*-glycan

Oligosaccharide	Name	Structure^a^	Relative activity^b^ (% ± S.D)
High mannose (Man6)	Man6		100
High mannose (Man9)	Man9		72.5 ± 17.8
Agalacto biantenna	A2		6.57 ± 1.35
Agalacto biantenna (+ bisecting GlcNAc)	A2B		*n.d*
Core-fucosylated agalacto biantenna	FA2		*n.d*
Agalacto (2, 4)-branched triantenna	A3		*n.d*
Agalacto (2,4/2,6)-branched tetraantenna	A4		*n.d*

a Circle, square, triangle, and PA represent mannose, GlcNAc, fucose, and 2-aminopyridine, respectively. Note that the structure of the proximal GlcNAc was altered upon PA-labeling.

b Relative activity is the mean and standard deviation from biological triplicates. The relative activity of human ENGase was calculated by setting the activity for Man6 as 100%. *n.d* represents not detected.

**Figure 2:**
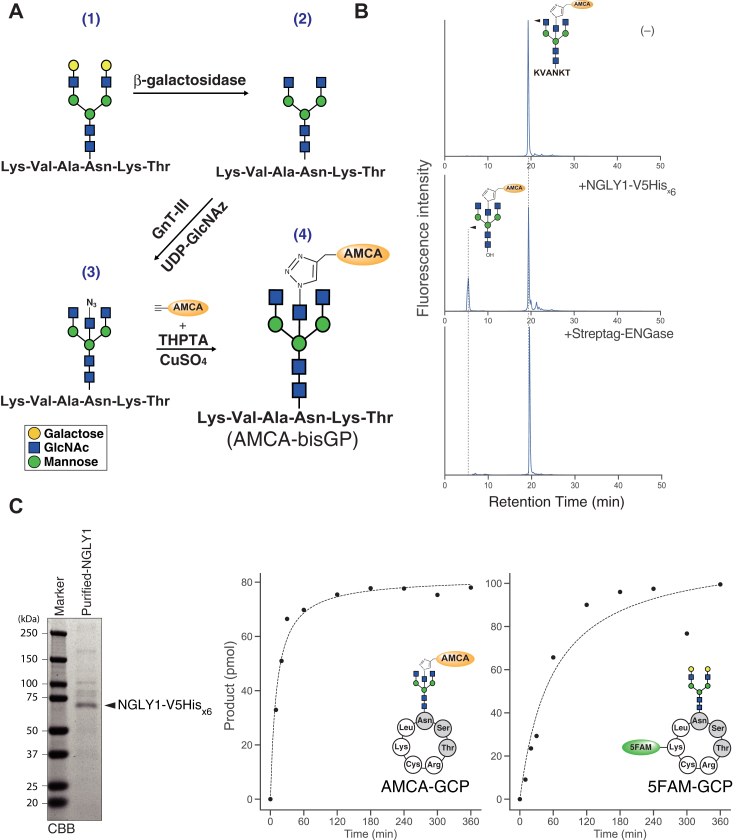
**Properties of glycopeptides carrying AMCA-labeled glycan.***A*, schematic representation of the chemoenzymatic synthesis of AMCA-bisGP. (1) commercially available asialo-glycopeptide was treated with β-galactosidase. (2) GlcNAz was transferred to the glycan by treating the glycopeptide with GnT-III and UDP-GlcNAz. (3) AMCA-alkyne was reacted with GlcNAz-labeled glycopeptides in THPTA solution supplemented with 20 mM CuSO_4._ (4) AMCA-bisGP was synthesized by click reaction. *B*, separation of AMCA-bisGP treated with or without deglycosylation enzymes (*i.e.*, purified NGLY-V5His_x6_ or Strep-tagged ENGase) at 37 °C overnight. *C*, *left panel* exhibits SDS-PAGE and CBB staining of the purified NGLY1-V5His_x6_ from extract of cell free expression system. *Right graphs* show reaction curves of the purified NGLY1-V5His_x6_, which is measured by two different substrates, AMCA-GCP and 5FAM-GCP. 5FAM-GCP, 5-carboxyfluorescein-labeled glycosylated hepta-cyclopeptide; AMCA, aminomethylcoumarin acetate; CBB, Coomassie brilliant blue; GCP, glycosylated hepta-cyclopeptide; GlcNAz, *N*-azidoacetylglucosamine; THPTA, Tris(3-hydroxypropyl-triazolylmethyl)amine.

Among the four glycan structures (*i.e.*, FA2, A3, A4, and A2B) that exhibit robust resistance to human ENGase, we selected the A2B form as a suitable glycan scaffold for a FRET-based NGLY1 probe for the following reasons. First, the transfer of *N*-azidoacetylglucosamine (GlcNAz), a crucial step for the chemoenzymatic integration of fluorophore through click chemistry, to A2 glycan for the generation of azide-labeled A2B appears to be the most straightforward among the four glycans. Namely, GlcNAz is easily transferred to the beta-linked mannose on the A2 glycan through a single reaction of the substrate with recombinant GnT-III and UDP-GlcNAz, both of which are commercially available (Fig. 2*A*). Second, NGLY1 is known to exhibit no deglycosylation activity for core-fucosylated glycans, so core-fucosylated substrate, such as FA2, is unlikely to be a suitable substrate for this enzyme (43). To determine whether the glycopeptide carrying fluorescence-labeled A2B exhibits resistance to human ENGase, we initially constructed a glycopeptide modified with aminomethylcoumarin acetate (AMCA)-labeled A2B, designated as an AMCA-labeled bisected *N*-glycopeptide (AMCA-bisGP) (Fig. 2*A*). After confirming the structure of the synthesized AMCA-bisGP (Fig. S1*B*), we studied its properties in detail. As shown in Figure 2*B*, AMCA-bisGP was deglycosylated by treatment with purified recombinant NGLY1, whereas no liberation of AMCA-labeled glycan was observed after treatment with purified recombinant ENGase. We also confirmed that, when an AMCA fluorophore was conjugated to the glycan, the substrate (AMCA-GCP) was found to be even a preferred substrate over 5FAM-GCP, in which Lys residue of the GCP is labeled with 5FAM (Fig. 2*C*). This result indicates that the AMCA-labeled A2B glycan is suitable for the FRET-based GCP probe.

### Validation of the FRET-based GCP probe for measuring NGLY1 activity

**Figure 3:**
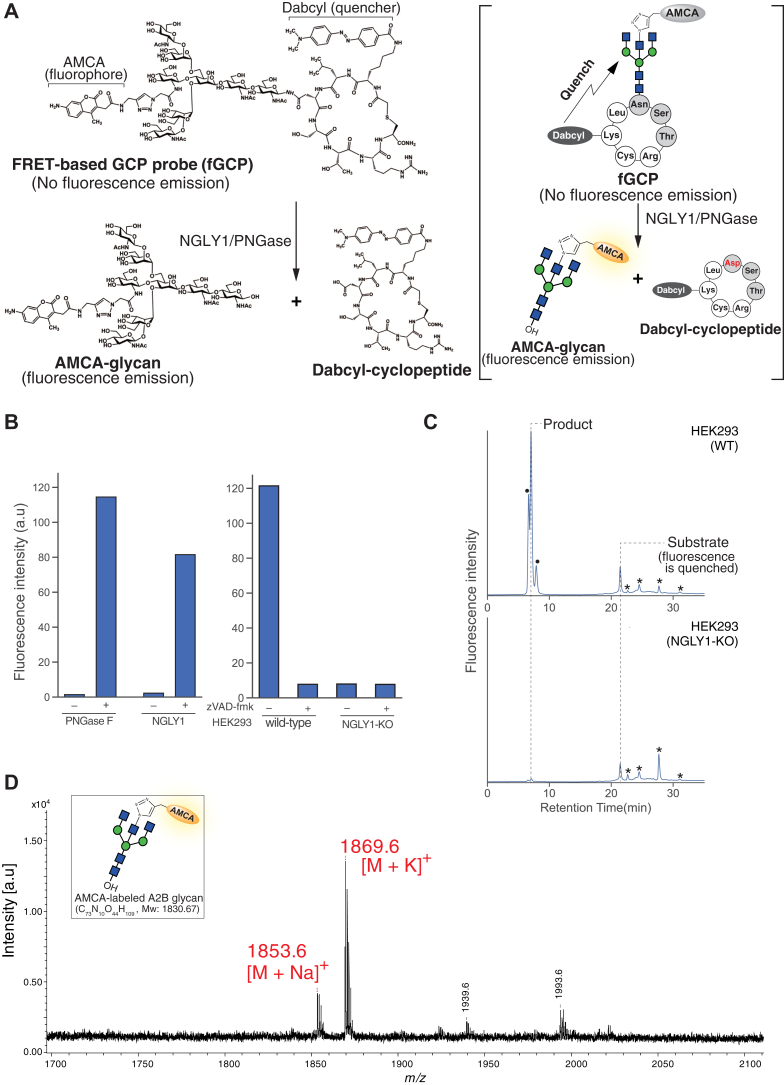
**fGCP can detect endogenous NGLY1 activity.***A*, structure and reaction mechanism of fGCP. The bisected-GlcNAc and lysine residues on glycosylated hepta-cyclopeptide were labeled with an AMCA fluorophore and a Dabcyl quencher, respectively. *B*, fluorescence intensity of fGCP incubated with various enzyme sources (PNGase F (positive control), NGLY1 or cell lysate) treated at 37 °C for 16 h with or without 100 μM of zVAD-fmk, a potent inhibitor for NGLY1 (45). The fluorescence intensity was measured by a microplate reader. *C*, HPLC separation of the substrate and product after reaction with HEK293 cell lysates. *Asterisks* and *closed circles* represent nonspecific and unidentified peaks, respectively. *D*, MALDI-ToF MS analysis of the reaction product separated and collected by HPLC in (*C*). AMCA, aminomethylcoumarin acetate; fGCP, FRET-based GCP probe; GCP, glycosylated hepta-cyclopeptide.

Having confirmed that AMCA-bisGP is a suitable substrate for NGLY1 but not for the cytosolic ENGase, we synthesized fGCP, in which the lysine residue was derivatized with a quencher, 4-((4-(dimethylamino)phenyl)azo)benzoic acid (Dabcyl), whereas the glycan was labeled with AMCA (Fig. 3*A*). We examined whether this probe could detect NGLY1 activity via fluorescence emission by measuring the fluorescence intensity of AMCA-glycans liberated from fGCP after incubating the substrate with crude cell lysate or pure deglycosylating enzymes (*i.e.*, NGLY1 or peptide:*N*-glycanase F (PNGase F) (44)). As shown in Figure 3*B*, an increase in fluorescence intensity was observed when fGCP was incubated with a purified enzyme. Additionally, strong fluorescence was detected after incubating fGCP with cell lysates derived from WT HEK293 cells, whereas no increase in intensity was observed with *NGLY1*-KO cell lysates or WT cell lysates treated with a potent NGLY1 inhibitor, zVAD-fmk (45) (Fig. 3*B*). Taken together, these results suggest that GCP produces no background signals through the formation of byproducts by endogenous ENGase or proteases. To further analyze the detailed structure of the released *N*-glycans, the reaction mixtures were separated by reversed-phase HPLC. As shown in Figure 3*C*, after incubating fGCP with cell lysates from WT HEK293 cells, we detected a peak for AMCA-labeled *N*-glycans that eluted at the same position as the authentic product. Notably, the peak intensity of deglycosylated fGCP was significantly higher than that of fGCP because the fluorophore was released from the quencher. However, no product formation was observed upon incubation of the fGCP with cell lysates derived from HEK293 *NGLY1*-KO cells. We also confirmed the molecular weight of the product peak (Fig. 3*C*; upper chart) by MALDI-TOF MS analysis and found it identical to the theoretical molecular mass of AMCA-labeled A2B glycan (Fig. 3*D*), suggesting that fGCP can detect NGLY1 activity even when crude cell lysates are used as enzyme sources.

### Real-time measurement of NGLY1 activity using fGCP

**Figure 4:**
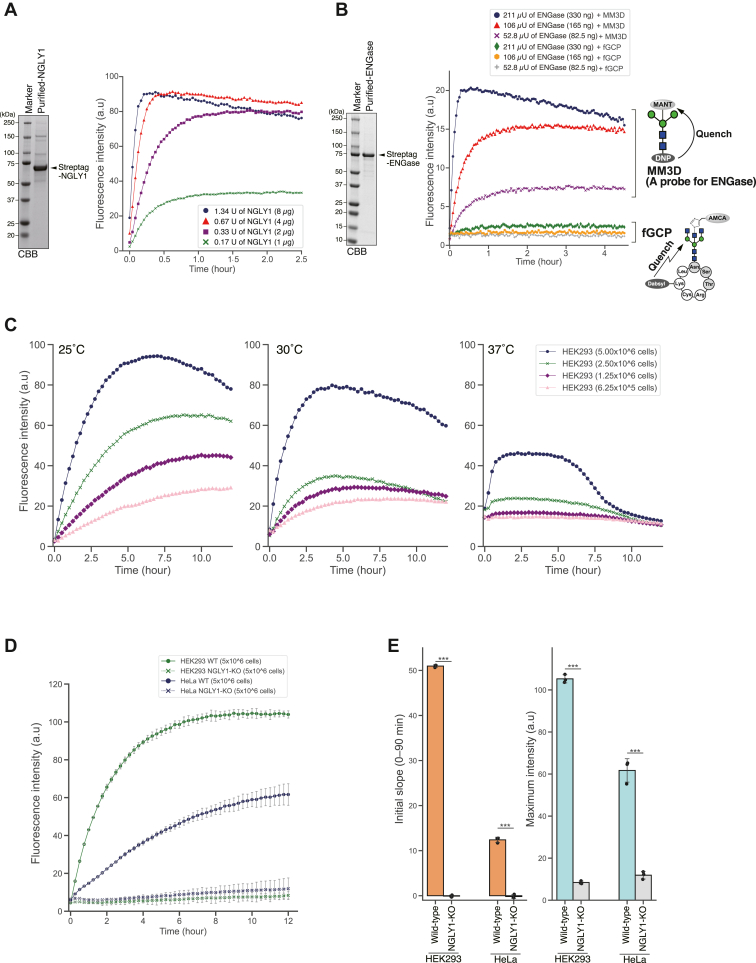
**Plate-based, real-time measurement of NGLY1 activity in various enzyme sources by fGCP.***A*, SDS-PAGE analysis of the purified strep-tagged NGLY1 (167 U/mg protein; *left panel*) from Expi293. Real-time measurement of NGLY1 activity (measuring every 2.5 min (*right panel*)) using fGCP with various concentrations of strep-tagged NGLY1 (1.3–0.2U) at 37 °C. The definition of the NGLY1 activity was described in “Purification of strep-tagged NGLY1 from Expi293 cells” in Experimental procedures. *B*, separation of the purified strep-tagged ENGase (0.64 U/mg protein) by SDS-PAGE (*left panel*). The activity of the purified strep-tagged ENGase (211–52.8 μU) was measured every 2.5 min by fGCP or MANT-M3GN2-DNP (MM3D), a FRET-based probe for ENGase (*right panel*) (39). The definition of the ENGase activity was described in “Purification of strep-tagged ENGase from HEK293 cells” in Experimental procedures. *C*, real-time measurement of NGLY1 activity (measuring every 15 min) in crude cell extracts prepared from various numbers of cells (6.25 × 10^5^–5.00 × 10^6^ cells) at three different temperatures (25 °C, 30 °C, and 37 °C). *D*, analysis of endogenous NGLY1 activity in HeLa and HEK293 cells. Each cell extract prepared from 5 × 10^6^ cells was incubated with fGCP for 12 h at 25 °C. The fluorescence intensity of fGCP was measured every 15 min. *E*, two parameters, initial slope (0–90 min) and max fluorescence intensity, of endogenous NGLY1 activity were measured in HeLa and HEK293 cells. Error bars are means ± S.D. from biological triplicates. For statistical analysis, a Student’s *t* test was applied. ∗∗∗, represents *p* < 0.001. fGCP, FRET-based GCP probe; GCP, glycosylated hepta-cyclopeptide.

Our next challenge was the plate-based, real-time measurement of NGLY1 activity from various enzyme sources using fGCP. We first established a reaction condition for real-time measurement of deglycosylation activity using purified Strep-tagged NGLY1 as an enzyme source. As shown in Figure 4*A*, we observed a concentration-dependent increase in fluorescence upon addition of Strep-tagged NGLY1. The reaction curve of NGLY1 with fGCP closely resembles those of previously reported substrates (37, 46). We also confirmed that fGCP has no detectable reactivity with purified Strep-tagged ENGase and is highly resistant to ENGase activity (Fig. 4*B*). This was shown by the comparison of the reaction curve of fGCP with that of MM3D, a FRET-based probe for ENGase (39). To achieve real-time measurement of endogenous NGLY1 activity in crude cell extracts using fGCP, we optimized the reaction temperature and protein concentration used in the assay. In a reaction at 37 °C, fluorescence intensity reduces significantly after prolonged incubation (6–12 h) possibly due to the complete loss of the reaction solution in each well resulting from evaporation (Fig. 4*C*, right graph). On the other hand, the reaction at 25 °C appears optimal (compared with 30 °C and 37 °C), though a slight reduction in fluorescence was observed after reaching the plateau (6–12 h) (Fig. 4*C*, left graph). This temperature was also optimal for reactions of fGCP with proteins in cell lysates from 5 × 10^6^ cells and produced the strongest fluorescence intensity and initial velocity (Fig. 4*C*, left graph).

### Evaluation of endogenous NGLY1 activity in various enzyme sources, including cell extracts from patients with NGLY1 deficiency

**Figure 5:**
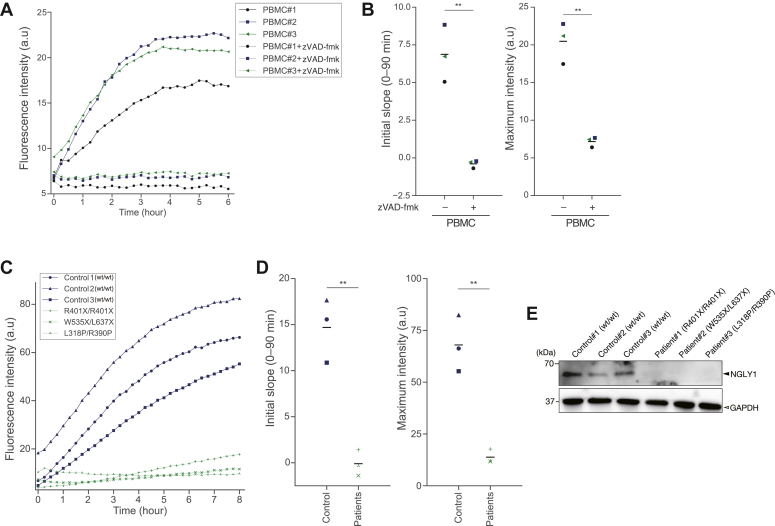
**Measuring the endogenous NGLY1 activity in PBMCs and fibroblasts.***A*, real-time measurement of endogenous NGLY1 activity (measuring every 15 min) in PBMCs with or without treatment with 100 μM of zVAD-fmk. Fluorescence intensity of each sample was measured by incubating fGCP with crude cell extract prepared from 5 × 10^6^ cells for 6 h at 25 °C. *B*, quantification of two parameters (*i.e.*, initial slope (0–90 min) and maximum fluorescence intensity) of (*A*). The *horizontal lines* represent means of biological triplicates (PBMC#1–3 with or without treatment of zVAD-fmk). *C*, real-time measurement of endogenous NGLY1 activity (measuring every 15 min) in fibroblasts. Fluorescence intensity of each sample was measured by incubating fGCP with crude cell extract prepared from 5 × 10^6^ cells for 8 h at 25 °C. *D*, quantitative analysis of endogenous NGLY1 activity in fibroblasts. The *horizontal line* represents the means of biological triplicates for controls' or patients’ derived samples. *E*, immunoblot analysis of NGLY1 in fibroblast cells derived from healthy subjects and three patients with NGLY1 deficiency. The blot was also probed with an anti-GAPDH antibody as a protein loading control. For statistical analysis, a Student’s *t* test was applied. ∗∗ represents *p* < 0.01. fGCP, FRET-based GCP probe; GCP, glycosylated hepta-cyclopeptide; PBMC, peripheral blood mononuclear cells.

In our previous study, we measured endogenous NGLY1 activity in various enzyme sources using an assay with 5FAM-GCP (Fig. 1*A*) (37). We further evaluated whether the FRET-based assay system could measure endogenous enzyme activities using enzyme sources previously assayed with 5FAM-GCP (37). The fGCP assay detected endogenous NGLY1 activity in WT cell lines (Fig. 4, *D* and *E*) and PBMCs (Fig. 5, *A* and *B*), whereas no activity was detected in *NGLY1*-KO cell lines (Fig. 4, *D* and *E*). Additionally, we found that endogenous NGLY1 activity was higher in HEK293 cells than in HeLa cells (Fig. 4*D*), which is consistent with previous observations of endogenous activity in these cells using 5FAM-GCP as a substrate (37). Next, we examined whether the fGCP assay could discriminate endogenous NGLY1 activity in the fibroblasts of NGLY1 deficiency patients from that of healthy control subjects. We detected no endogenous NGLY1 activity in the fibroblasts of three independent patients with mutant *NGLY1* alleles (Fig. 5, *C* and *D*), and NGLY1 protein was not detected in these samples by an anti-NGLY1 F(ab’)_2_ monoclonal antibody (Fig. 5*E*) that recognizes full-length NGLY1 protein and nonsense mutants (*i.e.*, truncated NGLY1) including the mutants used in this experiment (R401X, W535X, and L637X) (Fig. S3). These results suggest that NGLY1 activity was not detected in these patients’ cells because of the significant reduction of the level of mutant NGLY1 proteins by the premature decay of the mRNAs, as reported previously (37).

### Changes in endogenous NGLY1 levels in the aging rat brain

**Figure 6:**
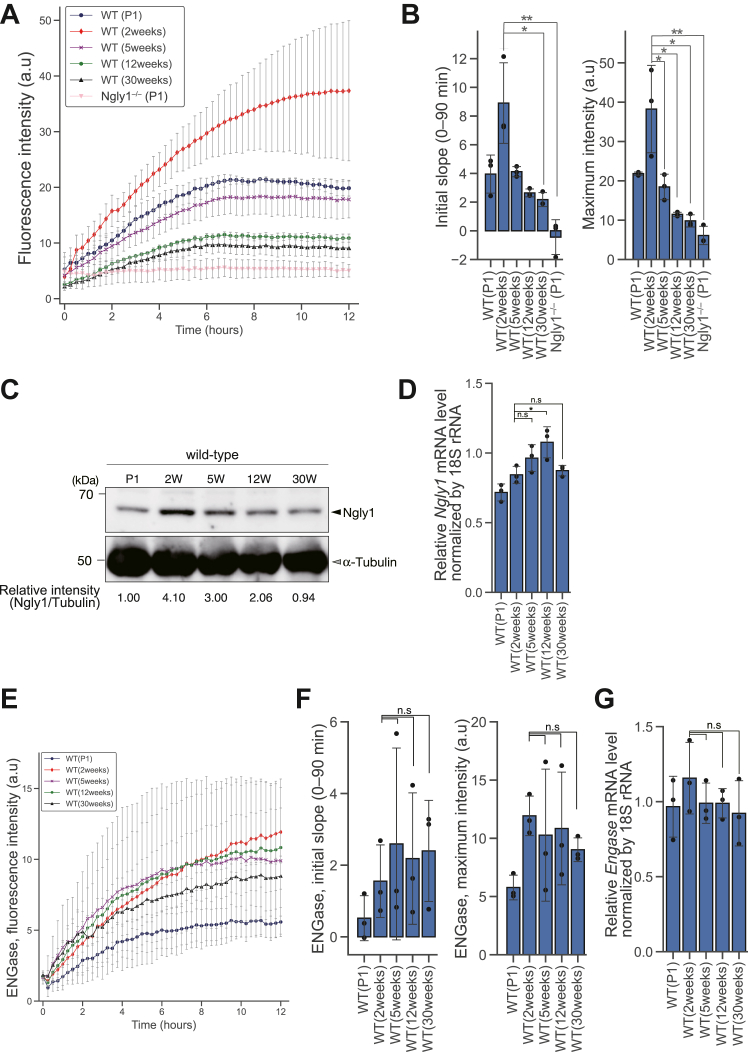
**Age-dependent changes of Ngly1 activity in the rat brains.***A*, real-time measurement of endogenous Ngly1 activity (measuring every 15 min) in rat brains of various ages. Fluorescence intensity of each sample was measured by incubating fGCP with 116 μg of protein extract from the brain for 12 h at 25 °C. *B*, quantitative analysis of (*A*). *C*, immunoblot analysis of NGLY1 in rat brains using anti-NGLY1 F(ab’)_2_. The blot was also probed with an anti-α-tubulin antibody as a protein loading control. The relative intensity of NGLY1 was calculated by normalizing the band intensity of NGLY1 with that of α-tubulin using ImageJ. *D*, quantitative real-time PCR analysis of *Ngly1* mRNA in the rat brain. The expression level of *Ngly1* was calculated relative to that of 18S ribosomal RNA. *E*, real-time measurement of endogenous ENGase activity (measuring every 15 min) in rat brains of various ages by assay with an MM3D probe. The fluorescence intensity of each sample was measured by incubating MM3D with 143 μg of protein extract from the brain for 12 h at 25 °C. *F*, quantitative analysis of (*E*). *G*, quantitative real-time PCR analysis of *Engase* mRNA in rat brains. The expression level of *Engase* was calculated relative to that of 18S ribosomal RNA. Error bars are means ± S.D. (n = 3, rats in each age). For statistical analysis, a Student’s *t* test was applied. *n.s, ∗*, and ∗∗ represent not significant, *p* < 0.05, and *p* < 0.01, respectively. fGCP, FRET-based GCP probe; GCP, glycosylated hepta-cyclopeptide; MM3D, MANT-M3GN2-DNP.

We previously reported that *Ngly1*^−/−^ rats exhibit neurological symptoms similar to those observed in patients with NGLY1 deficiency (32, 47). Notably, CNS neuroinflammation was prominent in *Ngly1*^*−/−*^ rats at 5 weeks of age but it was not observed in *Ngly1*^−/−^ rats at 2 weeks of age, while in WT rats, no neuroinflammation was observed at all ages tested (5–29 weeks) (47). This raises the possibility that endogenous Ngly1 activity within the CNS changes during the aging process. To investigate this hypothesis, we used the fGCP assay to measure endogenous Ngly1 activity in the brains of WT rats at various ages ranging from postnatal day 1 to 30 weeks. As shown in Figure 6, *A* and *B*, endogenous Ngly1 activity in the brain changed dramatically during the aging process, peaking at 2 weeks of age and then gradually declining. In contrast, the endogenous activity of the cytosolic ENGase (as measured by MM3D (39)) did not change significantly at any age tested (Fig. 6, *E* and *F*). Furthermore, no significant changes in *Engase* transcription were observed across all samples (Fig. 6*G*). Notably, the changes in endogenous Ngly1 activity were correlated with Ngly1 protein levels in the brain (Fig. 6*C*), but *Ngly1* transcription levels in the brain remained constant across all ages (Fig. 6*D*). These observations suggest that the aging process affects both intracellular Ngly1 activity and the abundance of Ngly1 protein, while not influencing the transcriptional level of *Ngly1* in the brain.

## Discussion

Previous studies have reported probes for detecting intracellular NGLY1 activity *in vivo*, including deglycosylation-dependent Venus (48) and a split-intein-based detection system (49). Methods for measuring PNGase/NGLY1 *in vitro* have also been developed using chemically modified substrates such as radioisotope-labeled glycosylated fetuin peptide and *S*-alkylated RNase B (38). Since the discovery of NGLY1 deficiency/NGLY1-related congenital disorder of deglycosylation in humans, a method has been needed for the facile and quantitative measurement of enzyme activity in various enzyme sources including crude cell extracts in order to facilitate molecular diagnosis. We previously succeeded in detecting endogenous NGLY1 activity in crude extracts of various cells and tissues using 5FAM-GCP, which is highly resistant to endogenous proteases/peptidases (37). However, this assay required HPLC separation and quantification of 5FAM-GCP, deglycosylated product, and byproduct (*N*-GlcNAc-GCP) (Fig. 1*A*). Here, we developed a novel FRET-based substrate, fGCP, for HPLC-independent detection of endogenous NGLY1 activity. This fluorescence-based assay system provides a facile NGLY1 assay method that is compatible with many standard clinical laboratories. We also demonstrated that fGCP can detect endogenous NGLY1 activity in various enzyme sources including cultured cells, human fibroblasts, PBMCs, and rat tissues, and can quantify endogenous NGLY1 activity by both the initial slope and maximum intensity of the reaction. We demonstrate that our plate-based fGCP assay can measure endogenous NGLY1 activity in 5 × 10^6^ PBMCs (Fig. 5*A*), indicating that this method is a promising tool for the diagnosis of NGLY1 deficiency and for evaluating treatment efficacy (*e.g.*, enzyme replacement therapy and/or gene therapy) in the near future. We have yet to successfully utilize this probe for real-time monitoring of intracellular NGLY1 activity in living cells by introducing fGCP into the culture medium. It is likely that the chemical properties of fGCP preclude its entry into cells, and future optimization of the structure of fGCP may overcome this issue by, for example, incorporating polylysine residues into the cyclopeptide to facilitate its endocytic transport (50).

In the reaction of 5FAM-GCP with endogenous NGLY1 in crude extracts, a byproduct (*N*-GlcNAc-GCP) is formed by ENGase, which is also present in the crude enzyme sources (Fig. 1*A*) (37, 38). The generation of *N*-GlcNAc-GCP is a major obstacle to developing 5FAM-GCP into fGCP because the ENGase-dependent cleavage of *N*-glycan on a FRET-based probe would also produce background fluorescence. Therefore, we examined the substrate specificity of the cytosolic ENGase in detail (Table 1). Specifically, we found that tri (2,4/2)- and tetra (2,4/2,6)-antennary glycans are highly resistant to human ENGase (Table 1), which is consistent with the observation that rat liver ENGase is not active against triantennary and tetraantennary glycans with a (2, 4)-branched structure (41). In addition, we discovered that a core-fucosylated glycan and a bisected glycan show certain resistance to human ENGase (Table 1), which is consistent with the substrate specificity observed for the fungal ENGase, Endo-Om (40). It has also been observed that bisected GlcNAc residues are poorer substrates for mammalian ENGase (41). On the other hand, it was reported that some GH85 ENGases, to which human ENGase belongs, can act on a fluorogenic probe of core-fucosylated glycan (51). Moreover, it has been well-documented that glycoproteins that have modified asparagines with core-fucosylated GlcNAc (GlcNAc(Fuc)-Asn; presumably a reaction product of ENGase), occur in human/mice cells/tissues, suggesting that mammalian ENGase can also act on core-fucosylated *N*-glycans (52, 53, 54). This seeming discrepancy may result from the disruption of the proximal GlcNAc structures by reductive amination with PA. Alternatively, weak activity of human ENGase toward core-fucosylated glycans could not have been detected in our assay system. Previous studies indicate that sialic acids present in the nonreducing end of *N*-glycans tend to reduce the deglycosylation activity of NGLY1 (43), suggesting that bulky molecules at the nonreducing end may obstruct NGLY1-dependent deglycosylation reactions. To assess the impact of the bulky fluorophore on NGLY1 activity, we compared GCP with a bulky AMCA fluorophore at the reducing end of glycan (AMCA-GCP), and 5FAM-GCP, where the Lys residue in GCP is labeled with 5FAM. We observed similar reaction curves for AMCA-GCP and 5FAM-GCP (Fig. 2*C*), indicating that the bulky AMCA group on GCP's bisecting-GlcNAc has no discernible effect on NGLY1 activity.

Assays for endogenous enzyme activity in crude samples often face issues with reaction interference via enzyme and substrate inactivation or degradation. Therefore, we optimized the reaction temperature to mitigate the inactivation of NGLY1 when crude cell extract was used as an enzyme source. We found that reaction of the substrate with crude cell extract at 25 °C led to an enzyme-dependent increase in fluorescence intensity (Fig. 4*C*, left panel), while reactions at 30 °C and 37 °C did not show this enzyme concentration dependence (Fig. 4*C*, middle and right panel). We used optimal fGCP assays conditions (25 °C; 5 × 10^6^ cells per lysate) to measure endogenous NGLY1 activity in human fibroblasts and in fibroblasts derived from three patients with NGLY1-deficiency. We found no detectable NGLY1 activity (Fig. 5, *C* and *D*) or NGLY1 protein (Fig. 5*E*) in the patients’ derived samples, which is consistent with previous observations that 24 recombinant NGLY1 mutant proteins, including the specific mutants expressed by these three patients, have no enzyme activity (37). The fibroblasts from two independent patients (*NGLY1*^*R458fs/R458fs*^ and *NGLY1*^*R401X/R401X*^) have both null NGLY1 activity and a significant reduction of *NGLY1* transcript levels resulting from the degradation of *NGLY1* mRNA by nonsense-mediated mRNA decay (37). Conceivably, the absence of NGLY1 proteins in these patients’ cells (*NGLY1*^*R401X/R401X*^*, NGLY1*^*W535X/L637X*^, and *NGLY1*^*L318P/R390P*^) may result from unstable mutant *NGLY1* mRNAs.

We also found that endogenous Ngly1 activity in rat brains changes during aging. Notably, Ngly1 activity increases in the first 2 weeks of age (Fig. 6, *A* and *B*), implying that Ngly1 may play a critical role in early brain development. Notably, this change in Ngly1 activity matches the change in Ngly1 protein levels (Fig. 6*C*), while *NGLY1* transcript levels remain unchanged (Fig. 6*D*). Further studies are required to understand the detailed mechanism by which Ngly1 protein levels change during aging. One potential explanation is that the stability and half-life of Ngly1 could be altered by increasing the sensitivity to some stresses caused by aging (*e.g.*, starvation, oxidative stress, endoplasmic reticulum stress, or heat stress and so on). Conversely, protein stability could be enhanced in 2-week-age rat brains due to the enhancement of stress-resistance by unknown mechanisms. An alternative possibility is that the decreased formation of the ternary complex of Ngly1 (p97-NGLY1-HR23B), which plays a crucial role in the efficient degradation of misfolded glycoproteins (2, 55, 56), might impact the stability of the Ngly1 protein. In any event, it should be noted that this analysis was made possible only by our facile, sensitive, fGCP-based assay, which is compatible with crude enzyme samples. NGLY1 research has been limited by the challenges of measuring NGLY1 activity in patient-derived cells, and this probe will be an invaluable tool for evaluating NGLY1 activity in patient-derived cells and for evaluating treatment efficacy after gene- or enzyme-replacement therapy.

## Experimental procedures

### Ethics statement

The experiments using PBMC from healthy subjects and primary fibroblasts were approved by the ethics committee of Takeda Pharmaceutical Company Limited (under permission numbers CS-00200736 and CS-00200187, respectively), and followed the principles outlined in the Declaration of Helsinki.

### Human cells

Three PBMCs from healthy subjects (20062287, 20062351, and 20062851) were purchased from HemaCare Corporation. Primary fibroblasts from healthy subjects (GM01652, GM03652, and GM23973) and three-independent patients (GM26586, GM26596, and GM26602) were purchased from Coriell Institute for Medical Research.

### Cell culture

Primary fibroblasts were cultured with Dulbecco's modified Eagle medium-low glucose (Fujifilm) supplemented with 10% fetal bovine serum (Cytiva; Hyclone) and antibiotics (Fujifilm). Other cell lines (HeLa and HEK293 cells) were cultured with Dulbecco's modified Eagle medium-high glucose (Fujifilm) supplemented with 10% fetal bovine serum (Cytiva) and antibiotics (Fujifilm). All cells were incubated at 37 °C in a humidified and 5% CO_2_ atmosphere.

### Animals

Care procedures and experiments of a rat outbred strain, Sprague–Dawley, conformed to the Association for Assessment and Accreditation of Laboratory Animal Care guidelines and were approved by the Experimental Animal Care and Use Committee of Takeda Pharmaceutical Company Limited. All rats were housed in individual cages in a room with controlled temperature (23 °C), humidity (55%), and lighting (lights on from 7:00 AM to 7:00 PM) and were fed a standard chow diet (CLEA Japan; CE2 diet) with free access to water.

### Construction of expression vectors

Double strands of Streptag-FLAG-human NGLY1 and Streptag-FLAG-human ENGase were chemically synthesized by Azenta. The sequence of these constructs was shown in Fig. S4. Each fragment was cloned into pcDNA3.4-TOPO according to the manufacturer's instructions. The construction of pEU-E01-NGLY1-V5His_x6_ was described previously (37).

### Expression and purification of NGLY1-V5His_x6_ from a cell-free system

Purification procedures of recombinant NGLY1-V5His_x6_ from wheat germ extract in a cell-free system (Protein Research Kit H (CellFree Sciences)) was according to the manufacturer's protocol. Briefly, 2 μg of a template plasmid, pEU-NGLY1-V5His_x6_, was incubated with transcription buffer for 2 h at 37 °C to transcribe the mRNA of NGLY1-V5His_x6_. After mixing the transcribed mRNA and wheat germ extract, the mRNA-wheat germ mixture was transferred to the bottom of the translation buffer to form a bilayer. The bilayered solution was incubated for 12 h at 15 °C for the translation reaction to proceed. NGLY1-V5His_x6_ in the crude reaction mixture was purified by Ni-Sepharose resin (Sigma-Aldrich Japan) as described previously (37).

### Purification of strep-tagged NGLY1 from Expi293 cells

Strep-tagged NGLY1 was expressed by transfection of pcDNA3.4-Streptag-FLAG-human NGLY1 with Expi293 (Thermo Fisher Scientific) according to the manufacturer's instruction. After transfection of the expression vector, the cells were cultured for 48 h and harvested. Then, the cell pellet was washed with PBS and dissolved in 50 ml of binding buffer (50 mM Tris–HCl [pH 8.0], 200 mM NaCl), including 1 mM DTT. The cells were lysed using a probe-type sonicator UD-21P (TOMMY) by five times of 60 s of sonication, separated by 60 s’ cooling periods on ice. The insoluble fraction was removed by centrifugation at 20,000*g* for 5 min at 4 °C, and the supernatant was further clarified by filtration using a syringe-filter unit (0.45 μm filter; Sigma-Aldrich Japan). The crude extract was incubated with strep–tactin resin (IBA Lifesciences GmbH) for 4 h at 4 °C with rotation. The resin with strep-tagged NGLY1 bound with them was washed (2 times) with strep–tactin wash buffer (100 mM Tris–HCl [pH 8.0], 150 mM NaCl, and 1 mM EDTA), including 1 mM DTT. The elution of strep-tagged NGLY1 was carried out with strep-tactin elution buffer (100 mM Tris–HCl [pH 8.0], 150 mM NaCl, 1 mM EDTA, and 2.5 mM desthiobiotin) (IBA Lifesciences GmbH). The eluted fraction was concentrated using Amicon Ultra-4 (molecular weight cut-off 30 k; Merck-Millipore). By the entire procedure, we obtained 1 to 2 mg of the recombinant NGLY1. This recombinant NGLY1 has 167 units activity per 1 mg protein activity (one unit of enzyme activity was defined as the amount of enzyme that catalyzed the release of 1 pmol of glycans from 5FAM-GCP at 37 °C per 1 min), which is slightly better than that of the purified NGLY1-V5His_x6_ expressed with cell-free system (32 units/mg protein) (37).

### Purification of strep-tagged ENGase from HEK293 cells

HEK293 cells were seeded in eight culture dishes (8 × 10^6^ cells/15 cm collagen-coated dishes; IWAKI Scitech). The seeded cells in the culture dishes were transfected with pcDNA3.4-Streptag-FLAG-human ENGase using FugeneHD (Promega). After culture for 48 h, cells were harvested and washed with PBS. The procedure of affinity-purification of strep-tagged ENGase was the same as that of strep-tagged NGLY1 (described above). By the entire procedure, we obtained 500 μg of the recombinant ENGase. This recombinant ENGase has 0.64 units activity per 1 mg protein (one unit of enzyme activity was defined as the amount of enzyme that catalyzed the release of 1 μmol of PA-GlcNAc from PA-labeled Man6GlcNAc2 at 37 °C per 1 min).

### Chemo-enzymatic synthesis of AMCA-bisGP

The scheme of enzymatic synthesis of AMCA-bisGP was shown in Figure 2*A*. A glycopeptide, Asialo-SGP (Lys-Val-Arg-Asn(glycan)-Lys-Thr), was purchased from Fushimi Pharmaceutical Ltd. For the modification of glycans on the glycopeptides from asialo-biantennary to asialo-agalacto-biantennary form, 100 μg of Asialo-SGP was treated with β-1,4-galactosidase (cloned from *Strepococcus pneumoniae* and expressed in *Escherichia coli*; Sigma-Aldrich Japan) in 50 mM sodium acetate buffer (pH 6.0) for 16 h at 37 °C. After denaturation of the β-1,4-galactosidase by boiling for 5 min, the resultant *N*-glycan (asialo-agalacto-biantenna form) on the glycopeptide was further modified with GlcNAz by reacting with 20 μg of purified recombinant human GnT-III (R and D systems) in the reaction buffer (25 mM Mes [pH 6.4], and 5 mM MnCl_2_) containing 50 nmol of UDP-GlcNAz (Chemily Glycoscience) for 16 h at 37 °C. For the confirmation of the structures of *N*-glycans modified with GlcNAz, *N*-glycans were liberated by PNGaseF (44), followed by labeling of the reducing end of each glucan with 2-aminobenzamide (AB) Glycan Labeling Kit (Ludger) according to the manufacturer's instruction. The structures of the AB-labeled glycan were determined by HPLC (as described in “High Performance Liquid Chromatography Analysis”) and LC-MS analysis (Fig. S1*A*). The authentic standard of AB-labeled glycans (asialo-biantenna (NA2), and agalacto-biantenna (A2)) were purchased from Ludger Ltd. A2 glycans modified with bisected GlcNAz was enzymatically synthesized by recombinant GnT-III using A2 form glycan and UDP-GlcNAc as a substrate (described above). The alkynylated-fluorescent probe (AMCA-alkyne; AAT Bioquest) was then conjugated with bisected GlcNAz on the glycopeptides by click-chemistry (57). Briefly, the dried-up glycopeptides modified with GlcNAz were resuspended in 150 μl of PBS containing 10 mM AMCA-alkyne. Then, 10 μl of 100 mM Tris(3-hydroxypropyl-triazolylmethyl)amine solution, 10 μl of 20 mM CuSO_4_ solution, and 10 μl of 300 mM sodium ascorbate solution were sequentially added to the resuspended glycopeptide, and the reaction solution was mixed well. The click reaction was performed for 30 min at room temperature under shaded conditions. After separating the reaction mixture by HPLC using Inert sustain C18 column (5 μm, 7.6 × 15 mm; GL Science), a PNGase F-sensitive peak was collected as the fraction of AMCA-bisGP (Fig. S1*B*; upper chart).

### Chemo-enzymatic synthesis of fGCP

#### Materials for chemical synthesis of fGCP

Glycosylated Fmoc-asparagine (Fmoc-Asn) derivative modified with A2-glycan was obtained from GlyTech, Inc. The following amino acids with protected side chains were utilized for the synthesis of the peptide: Ser/Thr(Bu^*t*^), Lys(Boc), Arg(Pbf), Asn(Trt), and Cys(Trt). Protecting groups, Bu^*t*^, Boc, Pbf, and Trt represent *t*-butyl, *t*-butoxycarbonyl, 2,2,4,6,7-pentamethyldihydrobenzofuran-5-sulfonyl, trityl, respectively.

#### Chemical synthesis of glycosylated hepta-cyclopeptide

The peptide chain was elongated on 100 μmol of Rink amide-ChemMatrix resin by a manual procedure using coupling with Fmoc-amino acid/*N,N*′-diisopropylcarbodiimide/1-hydroxybenzotriazole at a ratio of 5.0:5.0:5.0 (equivalents volume) in *N*-methyl-2-pyrrolidone (NMP) at room temperature for 30 min. Deprotection of *N*^α^-Fmoc groups was performed by treating the peptide with a 20% piperidine solution in *N,N*-dimethylformamide (DMF), with each treatment repeated twice for 5 min. During peptide synthesis, all washings after couplings and deprotections were performed with DMF.

Once the protected tetrapeptide (Ser-Thr-Arg-Cys) was constructed on the resin, the Fmoc-Asn residue modified with A2-glycan (Fmoc-Asn(A2)) was manually incorporated. The coupling reaction was performed for 2 h at room temperature in a solvent of Fmoc-Asn(A2)/3-(diethoxyphosphoryloxy)-1,2,3-benzotriazin-4(3*H*)-one (DEPBT)/DIPEA at a ratio of 1.5:4.5:4.5 (equivalents volume) in a mixture of NMP and dimethyl sulfoxide (DMSO) with a volume ratio of 1:1. For the subsequent manual synthesis, Fmoc-Leu and Fmoc-Lys(Boc) were sequentially coupled to the polypeptide by the incubation for 30 min at room temperature in a solvent composed of Fmoc-amino acid (Fmoc-Leu or Fmoc-Lys(Boc))/*N,N*′-diisopropylcarbodiimide/ethyl cyano(hydroxyimino)acetate (Oxima Pure) at a ratio of 5.0:5.0:5.0 (equivalents volume) in a mixture of NMP and DMSO with a volume ratio of 1:1.

After removing the Fmoc group from 100 μmol of glycopeptide resin by treatment with 20% piperidine/DMF, a chloroacetyl group was introduced to the *N*^α^-group of the peptide. This procedure was accomplished by treating the peptide resin with 0.17 g (1.0 mmol) of chloroacetic acid anhydride and 0.35 ml (2.0 mmol) of DIPEA in dichloromethane solution. The reaction mixture was agitated for 1 h at room temperature, and the resin was subsequently washed with DMF and dichloromethane, followed by dryness.

The peptide was then cleaved from the resin, and all protecting groups on the peptide were removed by treating it with 10 ml of a solution consisting of trifluoroacetic acid (TFA), triisopropylsilane, and water (in a ratio of 95:2.5:2.5, v/v/v) at room temperature for 1 h. The glycopeptide in the solution was precipitated by adding cold ethyl ether for partial purification. The resultant precipitate was dissolved in 50 ml of 0.1 M phosphate buffer (pH 6.8) containing 8 M guanidine hydrochloride and stirred for 1.5 h to convert the liner peptide to the cyclic peptide.

To remove the chloroacetyl groups from the hydroxyl groups of the A2 glycan, an aliquot of 10 ml of hydrazine hydrate was added to the reaction mixture and stirred for 30 min at room temperature. The pH of the solution was adjusted to 4.0 by adding acetic acid. The solution was then applied to a Sep-Pak Cartridge C18 for desalting, followed by further purification by reversed-phase HPLC with Proteonavi column (10 × 250 mm; Osaka Soda Co., Ltd). The structure of the final product was confirmed by electrospray ionization-mass spectrometry (ESI-MS). The entire procedure yielded 12 mg of the final product.

#### Labeling of the glycan moiety on GCP with AMCA

The modification of A2 glycan on 5 mg of GCP with GlcNAz by GnT-III was performed according to the procedure described in "Chemo-enzymatic synthesis of AMCA-bisGP". The reaction mixture was directly purified by the reversed-phase HPLC using DAISOPAK SP300-5-ODS-BIO columns (10 × 250 mm; Osaka Soda Co, Ltd). The conjugation of GlcNAz with AMCA was achieved through click chemistry as detailed in "Chemo-enzymatic synthesis of AMCA-bisGP". The reaction mixture was subsequently purified by a reversed-phase HPLC (Fig. S2*A*). The structure of the final product was confirmed by ESI-MS (Fig. S2*B*). The entire procedure yielded 2.7 mg of the final product.

#### Labeling of lysine residue on AMCA-GCP with Dabcyl

For the integration of Dabcyl into lysine residue on AMCA-GCP, 1.6 mg of AMCA-GCP, dissolved in 53 μl of DMSO, was added to a solution containing 2.2 mg of Dabcyl-*N*-succinimidyl ester, 53 μl of DMSO, and 0.8 μl of triethylamine. The mixture was stirred at room temperature for 2 h. For purification, the reaction mixture was diluted with 3 ml of 100 mM ascorbic acid containing 8 M guanidine hydrochloride and subjected to reversed-phase HPLC with an Osaka Soda DAISOPAK SP300-5-ODS-BIO column (10 × 250 mm) (Fig. S2*C*). The structure of the final product was confirmed by ESI-MS (Fig. S2*D*). The entire procedure yielded 1.1 mg of the final product.

### Activity assay of NGLY1 using 5FAM-GCP, AMCA-bisGP, or AMCA-GCP

The enzyme activity of purified NGLY1 and endogenous NGLY1 in cells/issues was measured as described previously (37). Briefly, the enzyme source was incubated in the NGLY1 buffer (50 mM of Tris–HCl [pH 7.5], 10 mM of sucrose, 5 mM of EDTA, 1 mM DTT, 1 mM of Pefabloc SC (Sigma-Aldrich Japan), and 1 × cOmplete protease inhibitor cocktail (Sigma-Aldrich Japan) containing 100 pmol of the substrate (*i.e.*, FAM-GCP, AMCA-bisGP, or AMCA-GCP) at 25 °C, 30 °C or 37 °C. The reaction was terminated by adding EtOH to a final concentration of 70%. The sample was evaporated to dryness in a SpeedVac concentrator and subjected to HPLC analysis as described in “High Performance Liquid Chromatography Analysis”.

### Activity assay of the endogenous NGLY1 using fGCP

For measurement of purified NGLY1, NGLY1 purified from Expi293 or cell-free system was dissolved in NGLY1 buffer, and 40 μl of NGLY1 solution was transferred to a 96-well black polystyrene microplate (clear flat bottom; Corning Inc). For the preparation of crude cell lysates, cultured cells (0.625–5.00 × 10^6^ cells) were washed twice with PBS and dissolved in 40 μl of NP40-lysis buffer (50 mM of Tris–HCl [pH 7.5], 0.5% (v/v) NP-40, 10 mM of sucrose, 5 mM of EDTA, 1 mM DTT, 50 μM Rabeprazole, 1 mM of Pefabloc SC, and 1 × cOmplete protease inhibitor cocktail) and lysed by incubation for 5 min on ice. Whereas, for the preparation of cell lysate from the whole brain of Sprague–Dawley rats, 25 to 50 mg of the brain was transferred to 1.5 ml of BioMasher II tube (Nippi, Inc) and resuspended in NGLY1 buffer. The tissue was lysed by 4 times 20 s of homogenization, separated by 20 s cooling periods on ice using PowerMasher II (Nippi Inc). The extract was centrifuged at 20,000*g* for 5 min at 4 °C to remove insoluble material, after which 40 μl of the supernatant, including 116 μg of proteins, was transferred to a 96-well black polystyrene microplate (clear flat bottom; Corning). Once adding 10 μl of 100 μM fGCP in the samples, NGLY1 activity was measured by detecting fluorescence (λexcitation; 353 nm, λemission; 450 nm, every 2.5 or 15 min with incubation at 25, 30, or 37 °C) using Varioskan LUX Multimode Microplate Reader.

### Activity assay of ENGase

For the activity assay of ENGase using PA-labeled glycan as a substrate, nonlabeled *N*-glycans (Man6, Man9, A2, A2B, FA2, A3, and A4) were purchased from Agilent Technologies, Inc. Preparation of the PA-labeled A2B glycan was performed by transferring bisecting GlcNAc to PA-labeled A2 glycan using recombinant human GnT-III (R and D systems) and UDP-GlcNAc (Sigma-Aldrich Japan) as described above. The structure of the synthesized A2B form glycan was confirmed by MALDI-ToF MS analysis. Pyridylaminaion of their reducing end was carried out as described previously (58, 59). To examine the substrate specificity for the various structures of substrates, the purified recombinant human ENGase was incubated with 50 pmol of each *N*-glycan in 50 μl of 100 mM Mes (pH 6.0) buffer for 5 min at 37 °C. The reaction was terminated by adding EtOH to a final concentration of 70%. The samples were evaporated to dryness in a Speed-vac concentrator and subjected to HPLC analysis (as described in “*High Performance Liquid Chromatography Analysis*”) for quantification of the reaction product. For the activity assay using a FRET-based probe, MM3D, the probe was purchased from TCI Chemicals. The assay was performed as described previously (39). Briefly, 40 μl of tissue extract, including 143 μg of proteins (prepared as described in “Activity assay of the endogenous NGLY1 using fGCP”), or purified ENGase was transferred to a 96-well black polystyrene microplate (clear flat bottom; Corning). Once adding 10 μl of 100 μM MM3D in the samples, ENGase activity was measured by detecting fluorescence (λexcitation; 353 nm, λemission; 450 nm, every 5 or 15 min with incubation at 25° or 37 °C) using Varioskan LUX Multimode Microplate Reader.

### High performance liquid chromatography analysis

Purification of AMCA-bisGP was performed by HPLC using InertSustain C18 column (5 μm, 7.6 × 150 mm; GL Sciences). For the purification of AMCA-bisGP, the mobile phases, eluent A (DW containing 0.1% TFA) and eluent B (100% acetonitrile containing 0.1% TFA) were prepared. The column was equilibrated with eluent A/eluent B (90/10) at a 1 ml/min flow rate. After injecting a sample, the unbound materials were washed with eluent A/eluent B (90/10) for 10 min. Then, AMCA-bisGP was eluted with eluent A/eluent B (85/15) and manually fractionated. AMCA-bisGP was detected by measuring fluorescence (λexcitation 346 nm, λemission 434 nm). For measurement of the enzyme activities, substrates, and reaction products were separated by HPLC using InertSustain C18 HP (3 μm, 3.0 × 150 mm, GL Sciences). For the separation of AMCA-bisGP and deglycosylated products, the column was equilibrated with eluent A/eluent B (95/5) at a flow rate of 0.45 ml/min. The concentration of eluent B was increased linearly from 5 to 95% over 40 min. AMCA-bisGP and its reaction products were detected by measuring fluorescence (λexcitation: 346 nm, λemission: 434 nm). The separation condition of 5FAM-GCP was performed as described previously (37). Briefly, the InertSustain C18 HP column (3 μm, 3.0 × 150 mm, GL Sciences) was equilibrated with eluent A/eluent B (75/25) at a flow rate of 0.45 ml/min. After injecting a sample, the concentration of eluent B was increased linearly from 25 to 70% over 25 min 5FAM-GCP and its reaction products were detected by measuring fluorescence (λexcitation: 494 nm, λemission: 518 nm). The separation of PA-labeling glycans was carried out using InertSustain C18 HP (3 μm, 3.0 × 150 mm; GL Sciences) and the mobile phases, eluent C (0.1 M ammonium acetate [pH 4.0]) and eluent D (0.1 M ammonium acetate containing 0.5% 1-butanol [pH 4.0]) as described previously (58). Briefly, the column temperature was set at 25 °C, and the flow rate was 0.45 ml/min. The column was equilibrated with eluent C/eluent D (95:5). After injecting a sample, the concentration of eluent D was increased linearly from 5 to 75% over 55 min. PA-glycans were detected by measuring fluorescence (λexcitation: 320 nm and λemission: 400 nm). For the separation of AB-labeled glycans, we used a Shodex NH2P-50 4E column (4.6-mm inner diameter × 250 mm; Shodex) and the mobile phases, eluent E (2% acetonitrile in methanol) and eluent F (5% acetonitrile and 3% triethylamine in water). The column temperature was set at 25 °C, and the flow rate was 1.0 ml/min. The gradient program was as follows: 0 to 2 min, 0 to 30% solvent F; 2 to 80 min, 30 to 95% solvent F; 80 to 85 min, isocratic 95% solvent F; 85 to 95 min, isocratic 0% solvent F. AB-labeled glycans were detected by measuring fluorescence (λexcitation: 330 nm and λemission: 420 nm).

### MALDI-ToF MS analysis

The detailed structure of AMCA-bisGP (Fig. S1*B*; lower spectrum) and the AMCA-labeled glycan liberated from fGCP (Fig. 3*D*) was confirmed by MALDI-ToF MS (autoflex speed; Bruker Daltonics) using 2,5-dihydroxybenzoic acid as a matrix at the Support Unit for Bio-Material Analysis in RIKEN CBS, Research Resources Division.

### Raising an antibody against human NGLY1

Screening of anti-NGLY1 antibody (His and Myc-tagged immunoglobulin G (IgG) F(ab’)_2_ antibody) against the purified strep-tagged human NGLY1 was performed by a phage display method using HuCAL PLATINUM library (Bio-Rad). Production of the anti-NGLY1 F(ab’)_2_ antibody was carried out by GeneFrontier Corporation. Specificity of this antibody was verified by immunoblotting of cell lysates extracted from NGLY1-KO HEK293 cells transiently expressing V5/His_x6_-tagged WT or mutant NGLY1 (Fig. S3).

### Immunoblotting

For immunoblotting of Ngly1 in rat brain, the tissue was lysed as described in “Activity assay of the endogenous NGLY1 using fGCP”. For immunoblotting of NGLY1 in fibroblasts, cells were grown on a 6 cm dish until subconfluent and harvested. After washing two times with PBS, cells were lysed by TBS-N buffer (10 mM Tris–HCl (pH7.5), 150 mM NaCl, 0.5% (v/v) Nonidet P-40, and 1 × cOmplete protease inhibitor cocktail (Sigma-Aldrich). The resulting cell extract was then centrifuged at 20,000*g* for 5 min at 4 °C to remove insoluble material, after which 6 × sample buffer (0.35 M Tris–HCl (pH 6.8), 10% SDS, 38% glycerol, 0.02% bromophenol blue, and 0.6 M DTT) was added to the supernatant. The samples were heated at 65 °C for 5 min. An aliquot of 25 μg of proteins was subjected to SDS–PAGE analysis using 4 to 20% polyacrylamide gel. The separated proteins in the gels were transferred to polyvinylidene fluoride membrane, and the membrane was blocked with TTBS (25 mM Tris–HCl (pH 7.4), 150 mM NaCl and 0.1% (v/v) Tween-20) containing 0.5% (w/v) skim milk. The blot was incubated with an anti-NGLY1 human antibody (1:2000 dilution), anti-tubulin mouse monoclonal antibody (1:10,000 dilution; Abcam), or anti-GAPDH mouse monoclonal antibody (1:5000; Merck-Millipore) followed by incubation with an anti-human IgG F(ab’)_2_ goat antibody conjugated with horseradish peroxidase (HRP) (1:2000 dilution; Jackson ImmunoResearch or an anti-mouse IgG antibody conjugated with HRP, (1:5000, Cell Signaling). Bands were reacted with Immobilon Western Chemiluminescent HRP (Merck-Millipore) and detected using ChemiDoc Touch MP (Bio-Rad).

### Real-time PCR

mRNA levels of *Ngly1* and *Engase* in rats’ brain were quantified by quantitative real-time PCR as described previously (37). Briefly, total RNA was extracted from the rats’ brain using ISOGEN II (Nippon gene). The extracted total RNA was further purified by RNeasy mini (Qiagen) and treated with RNaseFree DNase (Qiagen) to avoid contamination of genomic DNA. The complementary DNA was then generated using SuperScript VILO cDNA Synthesis Kit (Thermo Fisher Scientific) according to the manufacturer’s protocol. Real-Time PCR reactions were performed in TaqMan Universal PCR Master Mix (Thermo Fisher Scientific) with the TaqMan probes listed in Table S1. The expression levels of the target genes were measured in triplicate and normalized by the corresponding 18S ribosome RNA levels.

## Data availability

All data are available within the manuscript and supporting information.

## Supporting information

This article contains supporting information.

## Conflict of interest

The authors declare that they have no conflicts of interest with the contents of this article.
