# Supplementary material for: Development of a fluorescence and quencher-based FRET assay for detection of endogenous peptide:N-glycanase/NGLY1 activity
Source: J Biol Chem. 2024 Feb 28;300(4):107121. doi: 10.1016/j.jbc.2024.107121 (PMC11065741; doi:10.1016/j.jbc.2024.107121)
Supplement: Supplemental Figure 1–4 and Table 1 [file mmc1.docx]

**
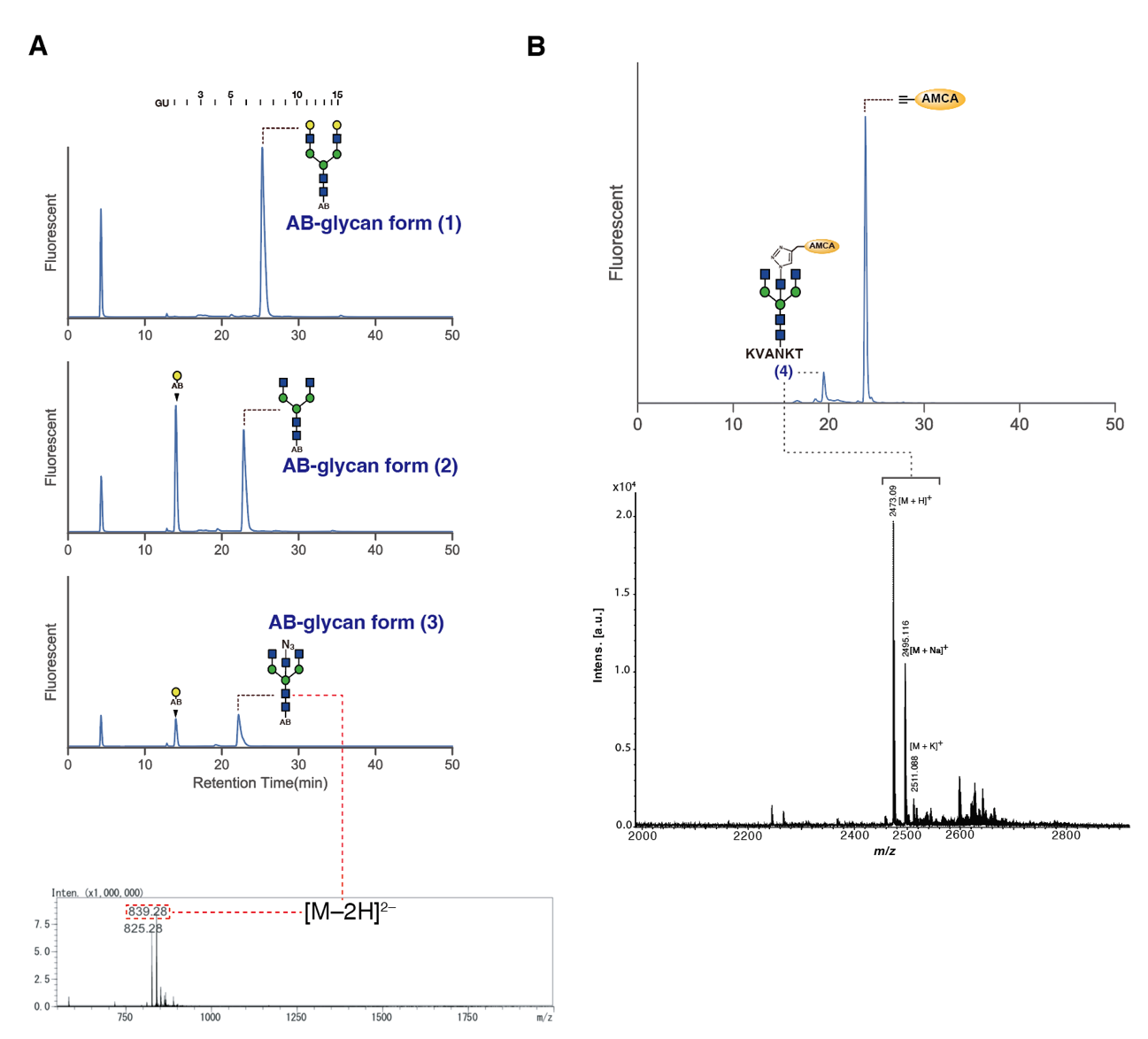
**

**Supplemental Figure 1:** **Structural confirmation of chemo-enzymatically synthesized AMCA-bisGP**

(A) HPLC analysis of glycans on product generated by each reaction shown in figure 2A. *N*-glycans were liberated by PNGase and labeled with 2-aminobenzamide (AB). Each peak was assigned by comparing the retention time of authentic standards. The peak of a glycan-transferred bisected GlcNAz was collected and further analyzed by LC-MS. The molecular weight of the AB-glycan form (3) was confirmed to be identical to the theoretical molecular weight. LC-MS spectrum recorded [M-2H]^2-^ ions (*m/z* 839.28). M represents molecular weight of the target glycan (C_65_H_104_N_10_O_41_). This analysis was performed on LCMS-IT-ToF (Shimadzu. Co. ltd, Kyoto, Japan) equipped with Nexcera UHPLC system (Shimadzu. Co. ltd) by electrospray ionization (ESI) negative ion mode [column: ACQUITY UPLC Glycan BEH Amide, 1.7 µm (2.1-mm inner diameter × 150 mm; Waters, Tokyo, Japan )]. (B) HPLC separation and MALDI-ToF MS analysis of the product (4). The molecular weight of the collected product of (4) was confirmed to be identical to the theoretical molecular weight. MALDI-ToF MS spectrum recorded [M + H]^+^ ions (*m/z* 2473.09), [M + Na]^+^ ions (*m/z* 2495.12), and [M + K]^+^ ions (*m/z* 2511.09). M represents molecular weight of the target glycopeptide (C_101_H_161_N_19_O_52_).


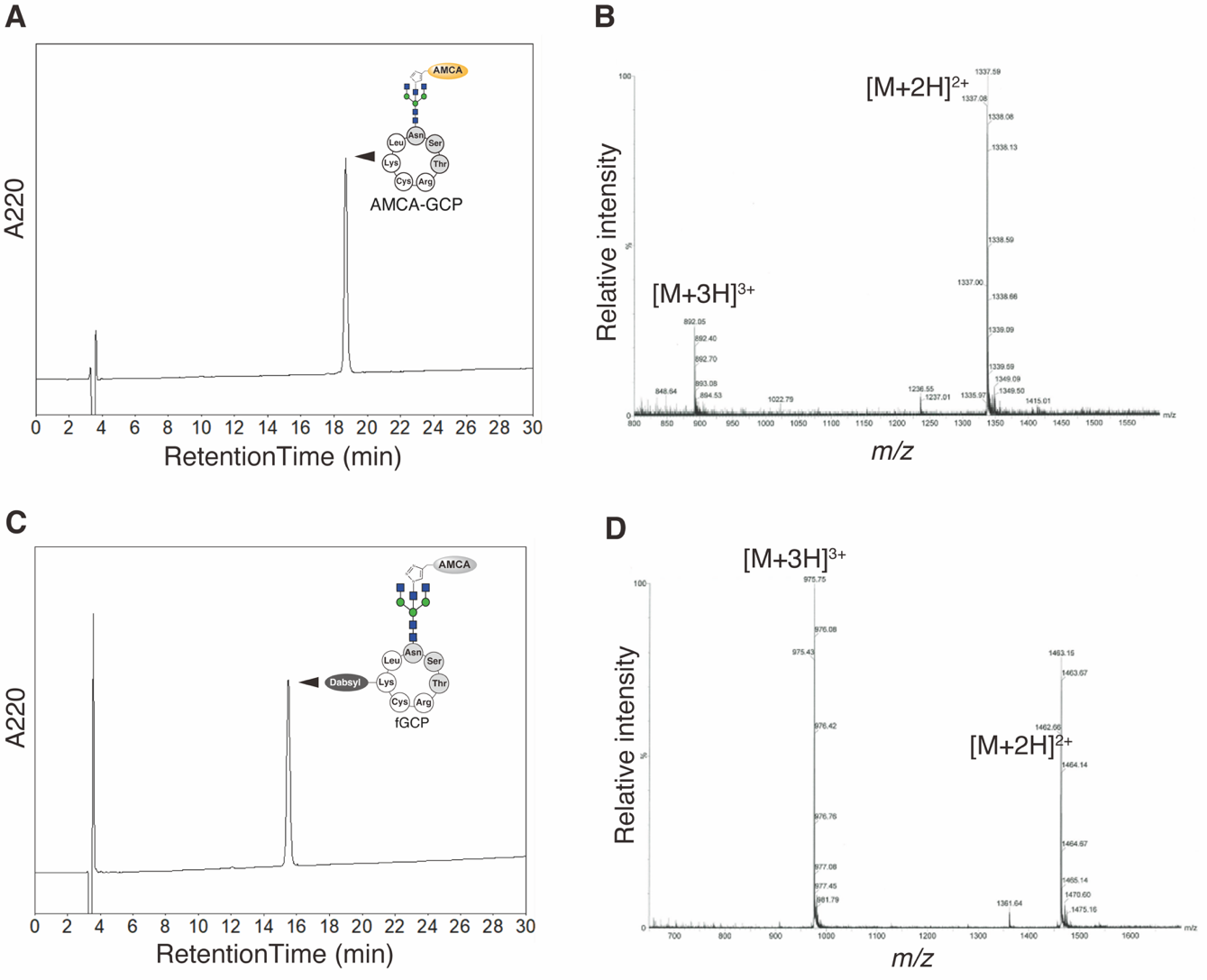


**Supplemental Figure 2:** **Structural confirmation of chemo-enzymatically synthesized AMCA-GCP and fGCP**

A) Separation of purified AMCA-GCP by reversed-phase HPLC using an Osaka Soda DAISOPAK SP300-5-ODS-BIO (4.6 × 250 mm). Mobile phase A (0.1% TFA in H_2_O) and mobile phase B (acetonitrile/H_2_O/TFA (90/10/0.09)) were prepared. For the analysis of AMCA-GCP, the concentration of mobile phase B was increased linearly from 5 to 20% over 30 min at a flow rate of 1.0 mL/min at 40˚C. (B) The molecular weight of the AMCA-GCP was confirmed to be identical to the theoretical molecular weight. ESI-MS spectrum recorded on [M + 3H]^3+^ (*m/z* 891.7) and [M + 2H]^2+^ ions (*m/z* 1337.1). M represents the target glycopeptide, AMCA-GCP (C_107_H_169_N_23_O_54_S). (C) Separation of purified fGCP by reversed-phase HPLC using an Osaka Soda DAISOPAK SP300-5-ODS-BIO (4.6 × 250 mm). For the analysis of fGCP, the concentration of mobile phase B was increased linearly from 20 to 40% over 30 min at a flow rate of 1.0 mL/min at 40˚C. (D) The molecular weight of the fGCP was confirmed to be identical to the theoretical molecular weight. ESI-MS spectrum recorded on [M + 3H]^3+^ (*m/z* 975.4) and [M + 2H]^2+^ ions (*m/z* 1462.7). M represents molecular weight of the target glycopeptide, fGCP (C_122_H_182_N_26_O_55_S).


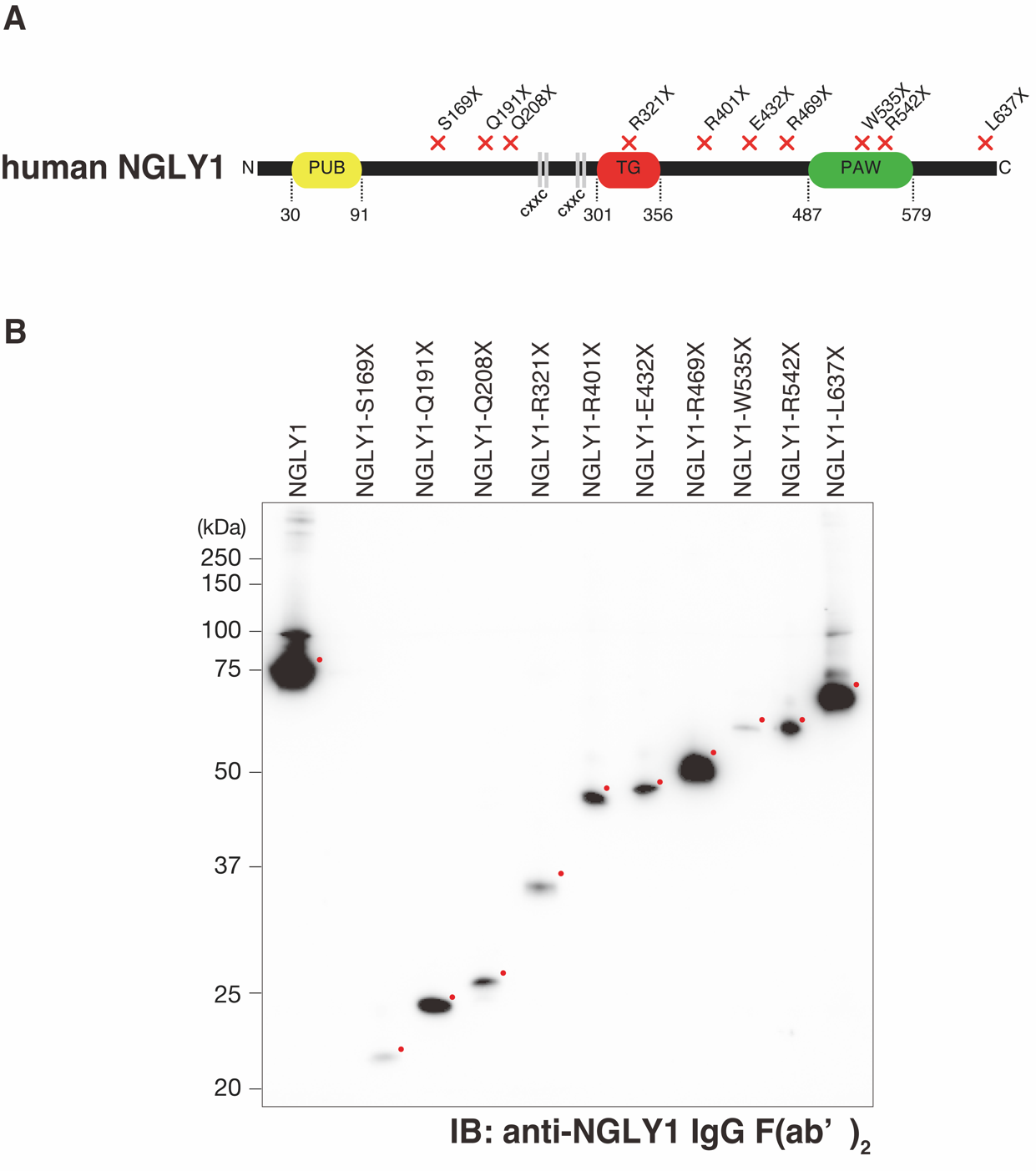
**Supplemental Figure 3: Evaluation of an anti-NGLY1 IgG F(ab’)_2_ antibody by immunoblot analysis.** (A) Schematic representation of primary structure of NGLY1 and nonsense mutation points identified from the patients. PUB, CxxC, TG and PAW represent **P**NGase- and **ub**iquitin-related domain, a putative zinc-binding motif, **t**rans**g**lutaminase domain and **p**resent in PNGase **a**nd other **w**orm proteins domain, respectively. (B) Immunoblot analysis of NGLY1 mutants expressed in the NGLY1-KO HEK293 cells. Wild-type and mutant NGLY1 were detected by anti-NGLY1 IgG F(ab’)_2_, which is originally generated. The bands of NGLY1 were marked with red circles. The difference in intensity for each band reflects the expression levels of the NGLY1 in NGLY1-KO HEK293 cells. **NGLY1**

GCCACCATGGCTAGCTGGAGCCACCCGCAGTTCGAAAAAGACTACAAGGATGACGATGACAAGGAGAATCTTTATTTTCAGAGCGGTGGTATGGCGGCGGCGGCATTGGGCAGCTCCTCAGGCTCGGCGTCCCCGGCCGTGGCTGAGCTCTGCCAGAACACCCCGGAGACCTTTTTGGAGGCCTCCAAGCTGCTGCTCACCTATGCTGACAACATCCTCAGAAACCCTAATGATGAAAAATATAGATCCATCCGGATTGGAAACACAGCCTTTTCTACTAGACTCTTGCCTGTCAGAGGAGCTGTTGAATGTTTATTTGAAATGGGCTTTGAAGAGGGAGAAACACATCTCATCTTTCCTAAAAAAGCTTCAGTGGAGCAGCTGCAAAAAATTCGTGACCTGATTGCCATAGAGAGAAGTAGCAGACTGGATGGCTCAAATAAGAGCCACAAAGTAAAGTCATCTCAGCAACCTGCAGCCAGTACCCAGCTTCCTACAACACCATCTTCAAATCCCAGTGGGTTAAACCAGCACACAAGGAACCGTCAAGGGCAGTCATCAGATCCACCATCTGCTTCAACGGTTGCTGCTGACTCAGCCATTCTAGAAGTTCTTCAGTCCAACATTCAGCATGTGCTGGTCTATGAAAATCCTGCTCTTCAGGAGAAAGCGTTGGCTTGTATTCCGGTCCAAGAACTAAAAAGGAAATCACAAGAAAAGTTATCGAGAGCTAGAAAATTGGATAAAGGTATCAATATAAGTGATGAGGATTTTCTTTTGCTGGAGCTTTTGCACTGGTTTAAGGAAGAATTTTTTCACTGGGTGAATAACGTTTTGTGCAGCAAATGTGGTGGACAGACTAGGTCTAGAGATAGATCATTACTGCCCAGTGATGATGAGCTGAAGTGGGGTGCAAAGGAAGTGGAAGATCATTACTGTGATGCCTGCCAGTTCAGCAATCGATTCCCAAGATATAATAACCCTGAGAAACTTTTGGAAACAAGATGTGGACGGTGTGGCGAGTGGGCCAATTGTTTTACACTGTGCTGCCGAGCTGTAGGGTTTGAAGCTCGCTATGTTTGGGATTACACAGACCATGTCTGGACAGAAGTCTATTCTCCTTCTCAGCAGCGGTGGCTGCACTGTGATGCATGTGAAGATGTCTGTGACAAGCCACTCCTTTATGAAATAGGATGGGGCAAGAAGCTTTCCTATGTCATAGCATTTTCAAAAGATGAGGTAGTTGATGTCACTTGGCGATATTCCTGCAAACATGAAGAGGTGATTGCCAGAAGAACTAAGGTTAAAGAAGCATTACTTCGAGACACTATTAATGGGCTTAATAAGCAGAGGCAACTGTTTTTGTCAGAAAACAGAAGGAAAGAACTTCTCCAGAGGATAATTGTGGAGCTTGTTGAATTTATATCTCCCAAAACCCCTAAACCTGGAGAACTTGGGGGAAGAATATCTGGGTCAGTGGCTTGGAGAGTAGCCCGAGGTGAAATGGGTCTACAGAGAAAAGAAACCTTGTTTATTCCCTGTGAAAATGAGAAGATTTCTAAACAGCTCCACCTTTGTTACAATATTGTGAAAGATCGTTATGTTCGAGTTTCAAATAACAATCAAACCATTTCTGGATGGGAGAATGGCGTGTGGAAAATGGAATCTATATTCAGAAAAGTTGAAACAGACTGGCACATGGTATATTTGGCCCGAAAGGAAGGATCATCTTTTGCTTATATTTCCTGGAAGTTTGAGTGTGGGTCAGTTGGCCTAAAAGTAGATAGCATTTCTATTAGAACAAGTAGTCAAACTTTTCAGACTGGAACAGTAGAATGGAAATTGCGATCTGATACAGCACAAGTAGAACTGACAGGCGATAACAGTCTTCACTCCTATGCTGATTTTTCTGGTGCCACTGAAGTTATTTTGGAAGCAGAATTAAGCAGAGGAGATGGTGATGTCGCTTGGCAACACACCCAGCTGTTTAGACAAAGCTTAAATGACCATGAAGAAAATTGTTTGGAGATAATTATAAAATTCAGTGACCTTTGA

NGLY1, Kozak sequence, Start codon, Strep-tag, FLAG, TEV site, Stop codon

**ENGase**

GCCACCATGGCTAGCTGGAGCCACCCGCAGTTCGAAAAAGACTACAAGGATGACGATGACAAGGAGAATCTTTATTTTCAGAGCGGTGGTATGGAGGCCGCTGCGGTGACAGTGACACGGTCTGCTACACGGCGGCGGCGGCGGCAACTGCAAGGACTGGCTGCTCCTGAGGCTGGAACACAGGAGGAGCAGGAGGATCAGGAGCCGCGGCCGCGGCGGCGGCGGCCGGGAAGGAGCATCAAAGATGAAGAAGAAGAGACAGTCTTTCGAGAGGTGGTCAGTTTTTCCCCGGACCCCCTGCCAGTTAGATATTATGACAAGGACACCACCAAACCAATCAGCTTTTACTTGTCTTCGCTGGAGGAGCTCTTGGCGTGGAAGCCCCGCTTGGAGGATGGCTTTAATGTGGCCCTGGAGCCCCTGGCGTGTCGCCAGCCCCCTCTGAGCAGCCAGAGGCCCCGGACTTTGTTGTGTCATGACATGATGGGCGGGTACCTGGATGACAGGTTCATTCAGGGCTCGGTGGTGCAGACTCCCTATGCTTTCTACCACTGGCAGTGCATCGACGTCTTTGTGTACTTCAGCCACCACACCGTCACCATTCCCCCAGTGGGCTGGACCAACACTGCCCACAGGCATGGGGTCTGCGTGCTGGGGACTTTCATCACGGAGTGGAATGAAGGGGGAAGGCTCTGTGAAGCCTTCCTGGCCGGGGATGAGCGCTCGTACCAGGCAGTGGCTGACCGGCTGGTCCAGATCACTCAGTTTTTTCGTTTTGATGGCTGGCTGATCAACATCGAGAACTCGCTGAGTCTGGCCGCTGTGGGGAACATGCCTCCTTTCCTGCGGTACCTCACCACACAGCTGCACCGGCAGGTCCCAGGGGGCCTGGTGCTCTGGTATGACAGCGTGGTGCAAAGTGGGCAGCTCAAATGGCAAGACGAACTCAACCAGCACAACAGGGTCTTCTTTGATTCCTGCGACGGCTTCTTCACTAACTATAACTGGCGGGAGGAGCACTTGGAGCGGATGCTGGGGCAGGCTGGGGAGCGCCGGGCTGATGTGTACGTGGGCGTGGATGTGTTTGCTCGAGGGAACGTGGTCGGAGGCCGATTCGACACAGACAAGTCGTTGGAGCTGATCCGAAAGCATGGCTTCTCCGTGGCTTTGTTTGCCCCCGGCTGGGTGTATGAGTGTCTGGAGAAGAAGGATTTCTTCCAGAACCAGGACAAGTTCTGGGGCCGACTGGAGCGTTATCTGCCCACACATAGCATCTGCTCCTTGCCTTTCGTCACGTCCTTCTGCCTGGGCATGGGTGCACGGAGGGTCTGCTATGGCCAGGAAGAGGCGGTAGGGCCCTGGTACCACCTGAGCGCCCAGGAGATCCAGCCCTTGTTTGGAGAACACAGGCTGGGAGGGGATGGCCGGGGCTGGGTGAGGACGCACTGCTGCCTGGAGGATGCCTGGCACGGAGGCAGCTCCCTGCTCGTCCGGGGTGTGATCCCACCGGAGGTTGGAAATGTGGCTGTGAGGTTATTTTCCCTGCAGGCCCCAGTGCCACCCAAGATTTACCTGTCCATGGTGTATAAGCTTGAGGGGCCCACGGACGTCACAGTTGCTTTGGAGCTGACCACAGGGGATGCCGGCAGCTGCCACATCGGTGGCATCTCAGTGTTGAACGCAGAAACAAGCTCAAGACACAGCCTCCGACCCCTCCGGGTGCCCCCCACCAAGCTGGCCAGATGGGTGGGCCGCTGCGGCCGGCAGCTGAGTGGGGGCTGGGTCCAGCACTGCTACGAGGTGAGCCTGCGTGGGTGCCTGCTGCTAGACCTCCTCGTTTGCTTCTCACGGCCGCCGGGTAGTCGGGAGGAGGAGAGCTTCACCTGTCGGCTTGGAGAGATCCAGGTGGTGGACGCTGCCAGCCTGCTGGCCCCTCTGCCCCAGGTGCAGGCCGTCACCATCTCTCACATCCGCTGGCAGCCATCCGCCTCTGAGCGGGAGGGGCCCCCTGCTCTGCTCCAGCTCAGCTGCACCCTGCACTGGTCCTTCCTCCTCTCACAAGTCCGTTGCTTCCGAATCCACTGCTGGGGAGGGATGAGTGATGACTCTCCGGGCAGGGAGCTGCCGAGGCCAGAGATGCCCATGTTCCTGGGGTTGGCTTTTGCCACCCAGTACCGGATAGTGGACCTGCTGGTGGAAGCCGCCGGGCCCGGCCAGGATCGTCGCATGGAATTTCTGGTGGAGCCTGTCCCCAAGGAAGGGTTCCGGGTACCTCAGGCCGAGTGGGGCAGGGCAGTTCTGCTTTATTCAGCCCCTGCATGA

ENGase, Kozak sequence, Start codon, Strep-tag, FLAG, TEV site, Stop codon,

**Supplemental Figure 4: The Sequences of chemically-synthesized NGLY1 and ENGase**

**Supplementary Table I: list of TaqMan® probes used in this study.**

| Symbol | Species | ID | Gene Name | Dye |
| --- | --- | --- | --- | --- |
| *Engase* | Rat | [Rn01751122_m1](https://www.thermofisher.com/taqman-gene-expression/product/Rn01751122_m1?CID=&ICID=&subtype=) | endo-β-*N*-acetylglucosaminidase | FAM |
| *Ngly1* | Rat | [Rn01756132_m1](https://www.thermofisher.com/taqman-gene-expression/product/Rn01756132_m1?CID=&ICID=&subtype=) | *N*-glycanase 1 | FAM |
| rRNA | S. cerevisiae, Human, Rat, Xenopus, Giardia, Mouse, Arabidopsis | [4308329](https://www.thermofisher.com/order/catalog/product/jp/en/4308329) | 18S ribosomal RNA | VIC |
| *Gapdh* | Rodent | [4308313](https://www.thermofisher.com/order/catalog/product/jp/en/4308313) | rodent *Gapdh* | VIC |
